# An Uncommon Case of Secondary Organizing Pneumonia Due to Influenza Type B

**DOI:** 10.3390/clinpract11010024

**Published:** 2021-03-10

**Authors:** Parth Shah, Philip T. Sobash, Krishna Vedala, Krishna Kakkera, Gilbert-Roy Kamoga

**Affiliations:** 1 Internal Medicine, White River Health System, Batesville, AR 72501, USA; pshah@wrmc.com (P.S.); kvedala@wrmc.com (K.V.); rkamoga@wrmc.com (G.-R.K.); 2 Pulmonology and Critical Care, White River Health System, Batesville, AR 72501, USA; kkakkera@wrmc.com

**Keywords:** secondary organizing pneumonia, influenza B, viral pneumonia

## Abstract

Secondary organizing pneumonia refers to a disease process caused by pulmonary tissue injury. Various insults can cause secondary organizing pneumonia, including multiple types of infections and cancer. The mainstay of diagnosis is a combination of imaging and lung biopsy showing inflammatory changes, specifically plugs with granulated tissue and fibrosis. Clinical suspicion needs to be raised for secondary organizing pneumonia when a patient is requiring increasing amounts of oxygen in the presence of treatment for pneumonia or another underlying lung disease. Here, we present the case of a 65-year-old male who presented with acute hypoxemic respiratory failure in the setting of previously having been tested positive for influenza B. Aggressive steroids with eventual tapering of his O_2_ requirements led to a successful outcome. While influenza has been reported as a cause of secondary organizing pneumonia after proceeding infection, these cases are usually represented by type A, rather than B.

## 1. Introduction

Organizing pneumonia (OP) is a clinicopathological syndrome that is comprised of systemic symptoms with consolidations on imaging with restrictive deficits on PFTs and granulation tissues found in the airways [1]. Organizing pneumonia can be categorized into either secondary organizing pneumonia (SOP) or cryptogenic if no known cause is identified (COP) [2]. Common symptoms of OP include fever, malaise, weight loss, dyspnea, and a persistent non-productive cough [3]. Onset often occurs in the 6th decade of life, with no gender bias [1]. Diagnosing OP requires both radiological and histopathological findings on lung biopsy demonstrating inflammatory plugs with granulation tissue embedded within connective tissue, known as Masson bodies. The mechanism of these changes is due to fibrosis of the affected alveolar ducts and alveoli. [4]. Tissue samples are often obtained with the assistance of video-assisted thoracoscopic surgery or transbronchial biopsy [4]. One particular hint for OP could be the lack of clinical improvement despite empiric antibiotic therapy [4]. Treatment varies based on the severity of the disease, with milder disease often not requiring any treatment at all [5]. Persistent and more fulminant disease, on the other hand, may require oral steroids tapered over the course of a few months [5]. If there is still a lack of improvement with steroids, cytotoxic therapy could also be utilized [3]. Prognosis for OP depends on the type, specifically with COP having a favorable prognosis, while SOP depends on the underlying cause [2]. There are many causes of SOP, such as drug-induced, bacterial, fungal, and viral, including COVID-19. One such virus that is well-documented is influenza type A [6]. Influenza type B is a less well-known and scantly reported cause of SOP presented in the literature [7]. 

## 2. Case Discussion

**Figure 1:**
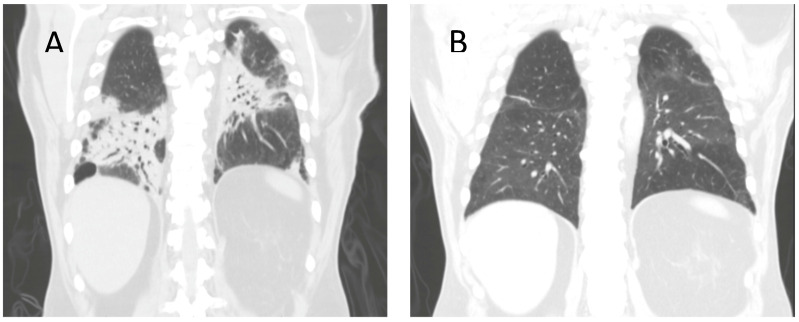
(**A**) CT Chest on admission showing significant multifocal airspace consolidation in mid-to-lower lung zones. (**B**) Repeat CT Chest after completion of steroid course, showing improved bibasilar consolidation.

We present the case of a 65-year-old male with no previous smoking or supplemental oxygen history who presented to the emergency department after a routine clinic visit that showed desaturation into the 70s. Past medical history was insignificant except for recent hospital admission the week prior that entailed a recent bilateral embolic stroke followed by a left carotid endarterectomy with 80% stenosis bilaterally, as well as a viral infection with influenza type B treated with Tamiflu. Initial evaluation upon admission revealed acute hypoxemic respiratory failure without hypercapnia. CT chest showed significant bilateral consolidation in the mid to lower lobes correlating with a bilateral pneumonia secondary to recent viral illness (Figure 1). Meropenem and Levaquin were started, followed by vancomycin as the patient continued to require more oxygen and non-invasive ventilation over the following three days. This included high-flow-nasal cannula (HFNC) initially, with advancement to Vapotherm to maintain the patient’s oxygenation status. Initial laboratory work revealed a white blood cell (WBC) of 13.3 (high of 23 during admission), erythrocyte sedimentation rate (ESR) of 88, and c-reactive protein (CRP) of 8.8. The respiratory viral panel was negative, and tests for other possible causes, including fungal and autoimmune causes, were negative. 

The patient underwent an endobronchial ultrasound (EBUS) bronchoscopy where the obtained samples were negative for source of infection from cytology, Gram stain, and sputum findings. The findings from EBUS bronchoscopy also included respiratory macrophages and inflammatory cells. Following this procedure, there was increasing concern for noninfectious organizing pneumonia as other diagnoses had been excluded. The patient was then started on IV steroids, and IV antibiotics were discontinued. In addition, nightly continuous positive airway pressure (CPAP) was added as there was also a concern of undiagnosed obstructive sleep apnea (OSA) as a contributing factor to the patient’s hypoxia. An echocardiogram with bubble study was additionally performed out of concern for possible shunting that could be worsening the patient’s oxygenation status, as the patient also had recent bilateral embolic stroke, but none was noted. The patient’s oxygen requirements began to slowly improve following this regimen over the course of several days, although an extended stay was required due to slow oxygen titration needs. An interval CT chest showed an improvement of consolidation as well. The patient’s oxygen status on the day of discharge remained at 2L on nasal cannula (NC) and he required supplemental oxygen upon discharge. The patient was also transitioned to per os (by mouth) (PO) steroids with instructions for slow taper upon discharge and to have close follow up with both his primary care provider (PCP) and pulmonologist, and referral for sleep study to evaluate for OSA workup. Follow-up CT chest after discharge showed significant resolution of consolidation (Figure 1).

## 3. Discussion

Our case represents a clinical scenario not widely reported in the literature with a complicated secondary organizing pneumonia due to influenza type B alone. It also emphasizes the importance of gathering any history relative to any preceding upper respiratory viral illness when suspecting pneumonia. The incidence of subsequent pneumonia in hospitalized patients has been shown to be 68.6% and 56.9%, respectively, for influenza types A and B [8]. Treatment with Oseltamivir alone has not been noted to prevent the development of SOP [9]. Early diagnosis and treatment with steroids is highly effective, but there can still be relapse after cessation [2]. While our patient presented in October 2019, it is not unreasonable in hindsight that this could still be a possible cause of his organizing pneumonia. This presentation was prior to the first positive COVID in the US, and before any testing was available. This is less likely though, as there was an objective cause that fit with his onset of symptoms. 

Asai et al. reported on a 23-year-old female without any relevant past medical history who had initially presented in the clinic for upper respiratory symptoms, but was then diagnosed with influenza B [7]. She failed to improve with Oseltamivir and developed respiratory failure, requiring 5 L supplemental oxygen [7]. She tested negative for any other pathogens including streptococcus and legionella [7]. She subsequently underwent transbronchial lung biopsy which confirmed lymphocytic alveolitis and SOP [7]. She was then started on corticosteroids and discharged with a 4-month taper [7]. Fortunately, she improved and did not have any recurrence after steroid cessation [7]. 

Kwok et al. reported a rare case of a 45-year-old female who developed SOP induced by influenza B and *S. pneumonia* co-infection [10]. She had a similar hospital course with respiratory failure, extended ventilatory support, and lack of improvement with antibiotic and antiviral treatment [10]. SOP was eventually diagnosed based on imaging and biopsy findings and was noted to have suffered substantial lung function impairment [10]. Steroids were initiated nearly 44 days after onset of infection. She was then treated with a 2-month taper of steroids and was noted to have made significant recovery in lung function nearly 13 months after her diagnosis [10]. Perhaps more importantly, the patient did not receive any influenza or pneumococcal vaccinations in the past, lending support to emphasizing the quadrivalent influenza vaccine covering 2 strains of Influenza B, instead of the trivalent vaccine which only covers 1 B strain [10]. 

Cornejo et al. presented two cases, one being a 52-year-old female smoker, while another was a 36-year-old diabetic male [6]. Although they had differing initial presentations, both patients required an extended length of time in the critical care unit, supplementary ventilation, and showed a lack of improvement with antibiotics alone [6]. Both also tested positive for a novel A H1N1 influenza and had confirmed histopathological evidence for SOP with open lung biopsy [6]. Their condition improved after receiving a high dose of steroids for an extended time [6]. Both cases confirm that SOP should be a differential when dealing with untreatable respiratory failure, and that open lung biopsy remains perhaps the most accurate choice for diagnosing SOP [6]. 

## 4. Conclusions

In cases of patients failing to make improvement despite empiric therapy, it is important to consider both common and uncommon etiologies for SOP. Workup, while extensive, should include preceding illnesses, malignancy, iatrogenic, autoimmune disorders, bacterial and viral panels. In addition, SOP also has a wide range of radiological findings, including focal consolidation, ground glass opacities, perilobular abnormalities, and reverse halo sign. Due to the complexity of diagnosis involving workup, careful attention must be given to similar radiologic findings to ensure correct diagnosis before treatment is initiated. 

## Data Availability

The data presented in this study are available on request from the corresponding author. The data are not publicly available in order to protect patient confidentiality.
